# Reversible Inactivation of the Higher Order Auditory Cortex during Fear Memory Consolidation Prevents Memory-Related Activity in the Basolateral Amygdala during Remote Memory Retrieval

**DOI:** 10.3389/fnbeh.2017.00138

**Published:** 2017-07-25

**Authors:** Marco Cambiaghi, Annamaria Renna, Luisella Milano, Benedetto Sacchetti

**Affiliations:** ^1^Rita Levi-Montalcini Department of Neuroscience, University of Turin Turin, Italy; ^2^Institute of Neuroscience Turin, Italy

**Keywords:** auditory cortex, amygdala, memory consolidation, memory storage and retrieval, neuronal synchrony

## Abstract

Recent findings have shown that the auditory cortex, and specifically the higher order Te2 area, is necessary for the consolidation of long-term fearful memories and that it interacts with the amygdala during the retrieval of long-term fearful memories. Here, we tested whether the reversible blockade of Te2 during memory consolidation may affect the activity changes occurring in the amygdala during the retrieval of fearful memories. To address this issue, we blocked Te2 in a reversible manner during memory consolidation processes. After 4 weeks, we assessed the activity of Te2 and individual nuclei of the amygdala during the retrieval of long-term memories. Rats in which Te2 was inactivated upon memory encoding showed a decreased freezing and failed to show Te2-to-basolateral amygdala (BLA) synchrony during memory retrieval. In addition, the expression of the immediate early gene zif268 in the lateral, basal and central amygdala nuclei did not show memory-related enhancement. As all sites were intact upon memory retrieval, we propose that the auditory cortex represents a key node in the consolidation of fear memories and it is essential for amygdala nuclei to support memory retrieval process.

## Introduction

Sensory stimuli that have acquired a threatening significance following an aversive experience are encoded in an intricate brain circuit composed of cortical and subcortical structures. The amygdala, a telencephalic region composed of several nuclei, is a crucial node of this network. Several cortical and subcortical areas interact with the amygdala during the encoding and retrieval of fearful memories. For instance, the amygdala exchanges information with the medial prefrontal cortex (mPF; Likhtik et al., 2014; Stujenske et al., 2014; Do-Monte et al., 2015; Karalis et al., 2016), which also plays an important role in the processing of emotional stimuli (Corcoran and Quirk, 2007; Likhtik and Paz, 2015; Dejean et al., 2016; Do Monte et al., 2016).

In addition to the mPF, the sensory cortex, such as the auditory cortex in the case of auditory stimuli paired to aversive events, is also involved in fearful memory processes. Originally, it was proposed that auditory stimuli were processed by the auditory cortex and the auditory thalamus, and subsequently auditory information reached the amygdala through both these pathways (LeDoux, 2000). However, many studies have indicated that the roles of the auditory cortex are more complex than the simple analysis and information transfer to the amygdala (reviewed in Weinberger, 2004, 2007, 2015; Fritz et al., 2007; Shamma and Fritz, 2014; Grosso et al., 2015a).

The rodents’ auditory cortex is subdivided into a central core and a surrounding belt region. The central area, corresponding to area Te1 of Zilles (1985), is assumed to be the primary auditory cortex, while the surrounding regions, (area Te2 and Te3 of Zilles) are considered higher order auditory cortices (Paxinos and Watson, 1986; Kolb and Tees, 1990). Te2 receives lighter projections from acoustic thalamic nuclei than the primary cortex (Paxinos and Watson, 1986; Kolb and Tees, 1990; Romanski and LeDoux, 1993), but it has heavier connections with the other neocortical areas and with subcortical nuclei, like the amygdala and the nucleus accumbens (Kolb and Tees, 1990; Romanski and LeDoux, 1993). The auditory cortex, whether primary or higher order, undergoes learning-evoked changes that occur shortly after training and that are still present during the retrieval of long-term fearful memories (Weinberger, 2004, 2007, 2015; Fritz et al., 2007; Shamma and Fritz, 2014; Grosso et al., 2015a). In addition, recent studies have shown that irreversible lesions (Sacco and Sacchetti, 2010; Grosso et al., 2015b; Cho et al., 2016) or reversible inactivation (Cambiaghi et al., 2015, 2016a) of the higher order auditory cortex Te2 when performed 4 weeks after training caused impairment of the long-term retention of auditory fearful memories, thus demonstrating that this cortex is necessary for the long-term storage/retrieval of fearful memories. The amygdala receives inputs from the auditory cortex, especially from the higher-order Te2 and Te3 areas (Romanski and LeDoux, 1993; Shi and Cassell, 1997; McDonald, 1998). More specifically, the Te2 cortex sends projections not only to lateral amygdala (LA) but also to basal amygdala (BA; Romanski and LeDoux, 1993; Shi and Cassell, 1997; McDonald, 1998). Remarkably, during the retrieval of long-term memories, Te2 activity is highly synchronized with the activity of the basolateral amygdala (BLA) in the theta frequency range (3–7 Hz), and a preponderant Te2-to-BLA directionality characterizes this dialog (Cambiaghi et al., 2016a); thus suggesting that Te2 leads BLA activity during fearful memory retrieval. Furthermore, Te2 blockade performed 1 day after training affected the retention of remote memories (Grosso et al., 2015b; Cambiaghi et al., 2016b). These results lead to the question of whether and how Te2 blockade performed during consolidation processes may affect Te2-to-BLA crosstalk and memory-related processes occurring in the amygdala nuclei during remote memory retrieval. The present study is aimed at clarifying these questions.

## Materials and Methods

### Subjects

Male Wistar rats (age, 65–80 days; weight, 250–350 g) were used. The animals were housed in plastic cages with food and water available *ad libitum*, under a 12 h light/dark cycle (lights on at 7:00 A.M.) at a constant temperature of 22 ± 1°. The animal sample size for each experiment was determined on the basis of our experience (Sacco and Sacchetti, 2010; Cambiaghi et al., 2016a, b) and of the current literature. All experiments were conducted in accordance with the European Communities Council 2010/63/EU and approved by the Italian Ministry of Health (Authorization No. 265/2011) and by the local Bioethical Committee of the University of Turin.

### Fear Conditioning Paradigm

#### Fear Memory Acquisition

A Skinner box module was employed as a conditioning chamber as in previous work (Cambiaghi et al., 2016a). The floor was made of stainless steel rods (1 cm in diameter, spaced 5 cm apart) connected to a shock delivery apparatus. The apparatus was enclosed within a sound attenuating chamber. Once inside, the animals were left undisturbed for 2 min. After this time, a series of seven auditory stimuli (8 s, 78 dB, 3000 Hz, 22-s intertrial interval) acting as conditioned stimuli (CSs) were administered. The last 1 s of each CS were paired with an unconditioned stimuli (US) consisting of a scrambled electric foot shock (intensity, 0.7 mA). Rats were left in the chamber for an additional 1 min, and then returned to the home cage. In the unpaired fear conditioning, seven pure tones (8 s 78 dB, 3000 Hz, 22 s intertrial interval) were delivered as CSs by a loudspeaker located 20 cm above the floor in a plastic box. Three hours after, animals were put in the conditioning chamber (see above) where seven shocks (1 s intensity, 0.7 mA) were delivered after 2 min every 30 s (Sacco and Sacchetti, 2010).

#### Fear Memory Retention

Four weeks after conditioning, the animals were handled for 2 days (5 min per day) before memory retention trial (Figure 2A). Memory was tested in a totally different apparatus located in a separate experimental room in order to avoid conditioned fear behavior to contextual cues (Sacco and Sacchetti, 2010; Cambiaghi et al., 2016a). The apparatus was a plastic cage with the floor and the sides made of transparent plastic and enclosed within a sound attenuating chamber equipped with an exhaust fan, which eliminated odorized air from the enclosure and provided background noise of 60 dB. Once inside, the subject was left undisturbed for 2 min. After this time, four CSs were administered identical to those used during conditioning. The rat behavior was recorded by means of a digital video camera. Freezing response was taken as a fear index and measured by means of a stopwatch by one person who did not know to which experimental group each animal belonged. Freezing was defined as the complete absence of somatic mobility, except for respiratory movements.

**Figure 1:**
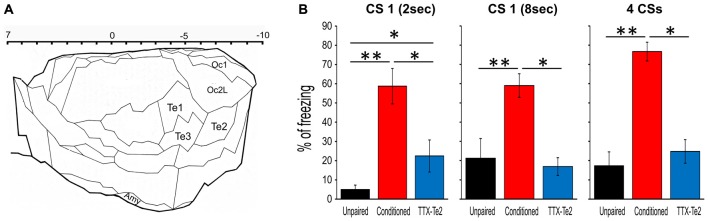
Reversible inactivation of the higher order auditory cortex Te2 impaired the expression of fear behavior during remote retrieval of auditory fearful memories. **(A)** Schematic representation of the secondary sensory cortices included in the present study. The upper scale indicates positive and negative distances from bregma. Plate adapted from Zilles (1985). **(B)** Percentage of freezing to the first 2 s of the first CS (left), the entire 8 s of the first CS (middle) and the overall four CSs (right) for the unpaired, conditioned and tetrodotoxin citrate (TTX)-injected rats. All data were mean ± SEM. Te1, primary auditory cortex; Te2 and Te3, secondary auditory areas; Amy, amygdala; Oc1, primary visual cortex; Oc2L, secondary visual areas (lateral). *P < 0.05; **P < 0.01.

**Figure 2:**
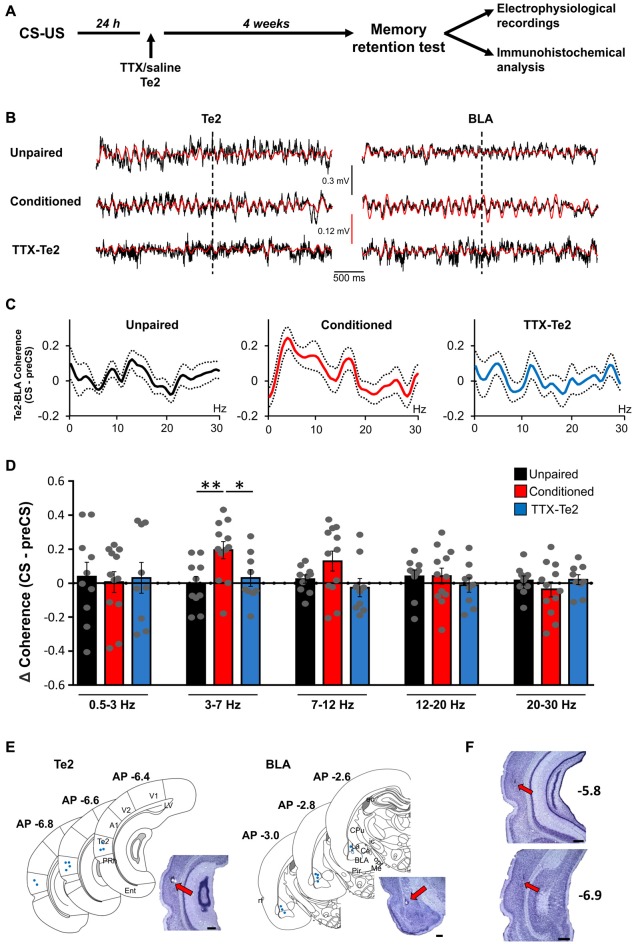
Te2 silencing upon memory consolidation prevented the increase in synchrony between Te2 and BLA activity during long-term memory retrieval. **(A)** Schematic diagram illustrating the experimental procedure employed to identify the role of Te2 in the retrieval vs. storage of fearful memories. **(B)** Representative local field potential (LFP) traces recorded in Te2 (left) and BLA (right) with their 3–7 Hz filtered signal superimposed (in red) around the first CS (dotted line). **(C)** Changes in the coherence between the BLA and the Te2 in unpaired (*n* = 10), conditioned (*n* = 12) and TTX-treated animals (*n* = 9) retrieving long-term memories. **(D)** Coherence between the Te2 and BLA was unchanged in TTX-treated animals (low-theta (3–7 Hz), 0.47 ± 0.29; high-theta (7–12 Hz), −0.18 ± 0.41) compared to unpaired animals (sign-rank, *P* = 0.179 and *P* = 0.199, respectively), but significantly lower relative to conditioned rats within the low-theta (sign-rank, *P* = 0.022). Representative histology of electrode tracks in Te2 and BLA of TTX-injected rats (*n* = 9) **(E)** and schematic localizations of electrode tips in the Te2 (left) and BLA (right). **(F)** Representative Nissl-stained sections showing that the administration of TTX into the Te2 cortex did not induce significant neuronal damage. The sections were taken at two different antero-posterior stereotaxic coordinates from the bregma. A1, primary auditory cortex; BLA, basolateral amygdala; Ce, central amygdala; CPU, caudate putamen (striatum); ec, external capsule; Ent, entorhinal cortex; ic, internal capsule; La, lateral amygdala; LV, lateral ventricle; Me, medial amygdala; opt, optic tract; Pir, piriform cortex; PRh, perirhinal cortex; rf, rhinal fissure; Te2, secondary auditory cortex; V1 and V2, primary and secondary visual cortices. Plates are adapted from the atlas of Paxinos and Watson (1986). Te2 was defined according to the atlas of Zilles (1985). Scale bars, 500 μm. *P < 0.05; **P < 0.01.

### Surgical Procedures

Reversible functional inactivation of the secondary auditory cortex Te2 was induced by the bilateral administration of Tetrodotoxin citrate (TTX) at the following stereotaxic coordinates: anteroposterior (AP), −5.8; lateral (L), ±6.5; and ventral (V), 6.0, 0.5 μl; and (AP), −6.8; (L), ±6.5 and (V), 6.0, 0.55 μl (Cambiaghi et al., 2016b). TTX (10 ng/μl) was dissolved in saline and then injected 24 h after training. Control subjects received saline solution instead of TTX. Rats were mounted in the stereotaxic apparatus (ear bars, 45°), an incision of the skull was made, and small burr holes were drilled to allow the penetration of a 28 gauge infusion needle. A 10 μl Hamilton syringe mounted on an infusion pump was used to deliver infusions at a rate of 0.25 μl/min. The needle was left in place for another 1 min. Incisions were closed (stainless steel wound clips), and animals were given s.c. injections of the analgesic/anti-inflammatory ketoprofen (2 mg/kg). Rats were kept warm and under observation until recovery from anesthesia.

### *In Vivo* Electrophysiology

For recording of extracellular field potentials, stainless steel wires were implanted unilaterally (right side) at least 1 week before memory recall. Electrodes were built with three stainless steel wires (Ø 150 μm) to ensure mechanical stability of the bundle (to obtain straight trajectory in the brain tissue), and they were connected to a 10-pin connector (Omnetics). Under deep anesthesia, electrodes were stereotaxically implanted in BLA and Te2, according to the following coordinates, in mm: BLA, AP = −2.7, *L* = 5.0, *V* = 8.1; Te2, AP = −6.6, *L* = 6.5–6.7, *V* = 6.0. A silver wire over contralateral parietal areas served as reference and ground. All implants were secured using Ketacem cement. All recordings were performed in a customized Faraday chamber. Local field potentials (LFPs) were recorded (Plexon acquisition system, 16-channel) and initially digitalized at 1 kHz and stored on a hard drive for offline analysis. The LFPs were very similar across the three channels belonging to the same bundle. At the conclusion of each experiment, animals were transcardially perfused for the detection of the recording sites throughout Nissl staining.

LFPs epochs were visually examined and *power spectra* of artifact-free segments were computed using fast Fourier transforms by using the commercial software NeuroExplorer with a 0.25 Hz resolution. Mean power spectra were divided into five frequency bands: *delta* (0.5–3 Hz), *low-theta* (3.01–7 Hz), *high-theta* (7.01–12 Hz), *beta1* (12.01–20 Hz) and *beta2* (20.01–30 Hz). Baseline was evaluated by averaging three 2-s epochs within the pre-CS period. For the 8 s analysis we averaged four windows of 2 s length with 0% overlap. Relative power was calculated by dividing the absolute amplitude within the aforementioned frequency ranges by the corresponding measures of total amplitude. Spectrograms were calculated using the software NeuroExplorer.

The *coherence* between LFP channels was measured by magnitude squared coherence (MSC), using the function *mscohere* in *Matlab* signal toolbox, which is a coherence estimate of the input signals *x* and *y* by using Welch’s averaged, modified periodogram method. The MSC estimate is a function of frequency with values between 0 and 1 and indicates how well *x* corresponds to *y* at each frequency. Segments of 2 s duration are split into 8 epochs with 50% overlap. The MSC estimate is calculated over the frequency range of 0.5–30 Hz for each rat. Difference in coherence were obtained by subtraction of coherence values (CS−preCS) and statistics were performed on the average difference in coherence within the frequency bands of interest. PreCS was calculated by averaging five 2 s epochs within the entire pre-CS period (Cambiaghi et al., 2016a).

### Zif268 Protein Expression Analysis

As in our previous study (Sacco and Sacchetti, 2010), 4 weeks after training, all animals were handled and habituated to the new cage for 5 days, each day for 7 min. The cage was different from that employed during conditioning. The sixth day, all groups were exposed to the acoustic stimuli (see “fear memory retention” paragraph). Freezing was used as an index to measure fear conditioning. Ninety minutes after the completion of memory retention test, rats were deeply anesthetized and perfused intracardially with 4% paraformaldehyde. The brains were dissected, stored overnight at 4°C, and finally transferred to 30% sucrose. Coronal sections (50-μm) were cut on a vibratome and collected in PBS. Free-floating sections were pretreated with 0.3% H_2_O_2_ in PBS to reduce endogenous peroxidase activity. After four rinses, sections were incubated in a blocking solution (2% bovine serum albumin (BSA), 2% normal goat serum and 0.2% Triton X-100) for 1 h at RT). Then, they were incubated in primary polyclonal rabbit anti-zif268 (1:2000 dilution, Santa Cruz) antibodies in the blocking solution overnight al the RT. Subsequently, sections were washed with PBS and incubated for 2 h at RT with biotinylated goat antirabbit IgG (1:2000 in PBS, Jackson Laboratories) followed by 1 h at RT in ABC. Sections were rinsed in PBS. The peroxidase reaction end-product was visualized by incubating sections in 0.05 M Tris (pH 7.6) containing 3.3′ DAB (0.037%) as chromogen and hydrogen peroxide (0.015%) for 5 min. Finally, immunolabeled sections were washed in PBS, mounted on gelatin-coated slides, dehydrated and coverslipped. The slices were analyzed using Neurolucida software connected to a microscope via a color CCD camera. The quantification of zif268-positive cells was carried out at X 10 magnification. Immunoreactive nuclei were counted bilaterally using at least three serial sections for each area without experimenter knowledge of the experimental condition. The number of nuclei expressing zif268 was quantified in the area of interest at the coordinates: lateral and basal nuclei of the amygdala AP = from −2.5 mm to −3.3 mm; central amygdala AP = from −2.0 mm to −2.8 mm (Sacco and Sacchetti, 2010). The mean count of each animal was divided by the mean count of the respective naïve control group in order to generate a normalized count for each animal. Data were then averaged in order to produce the mean of each group.

### Histology

The needle track in the case of TTX-injections was histologically verified at the end of the experiments with Nissl staining, using the conventional procedure (Sacco and Sacchetti, 2010).

### Statistical Analysis

Student’s *t*-test was used for comparing freezing responses. One-way ANOVA and Newman-Keuls multiple comparisons test were employed to compare Zif268 protein levels in the different behavioral groups. Nonparametric Kruskal-Wallis test followed by *Wilcoxon* signed-rank *test* were employed for the electrophysiological analysis. Experiments were replicated in two (for electrophysiological recordings) and in three (for immunohistochemical analysis) independent trials. All results were reported as means with Standard Error Mean (SEM) as indicated in figure legends.

## Results

### Reversible Blockade of Te2 during Fear Memory Consolidation Prevents Te2-BLA Interplay during the Retrieval of Long-Term Fearful Memories

To establish the effects that Te2 blockade performed during the consolidation of auditory fear memories may have on Te2-to-BLA crosstalk on BLA activity occurring during memory retrieval process, we reversibly blocked the Te2 at 1 day after training and we subsequently tested long-term memory retention and Te2-BLA interplay 1 month later (Figures 1, 2A). As in our previous studies (Grosso et al., 2015b; Cambiaghi et al., 2016a), in order to reversibly block Te2 during memory consolidation processes, we administered Tetrodotoxin (TTX), a voltage-dependent sodium channels blocker. Unlike optogenetic manipulations or muscimol, TTX blocks neural activity for several hours (i.e., for at least 6–8 h; Zhuravin and Bures, 1991; Ambrogi Lorenzini et al., 1999; Martin and Ghez, 1999), and it is therefore suitable for interfering with long-term memory consolidation processes that require fast synaptic transmission for several hours and days (Ambrogi Lorenzini et al., 1999; Riedel et al., 1999; Sacchetti et al., 1999a, 2002; Lesburguères et al., 2011). Moreover, TTX has fully reversible effects and does not induce any permanent damage (Ambrogi Lorenzini et al., 1999; Sacchetti et al., 1999a, 2002). To interfere with long-term system consolidation but not with the cellular consolidation mechanisms that are triggered immediately after training, TTX was administered 1 day after learning (Lesburguères et al., 2011; Cambiaghi et al., 2016b).

Rats were trained to associate acoustic stimuli CSs with aversive ones USs. Long-term memory retention was assessed 1 month later by measuring freezing behavior elicited by CSs previously paired with the US (Sacchetti et al., 1999b; Sacco and Sacchetti, 2010; Grosso et al., 2015b). In line with our previous studies (Grosso et al., 2015b; Cambiaghi et al., 2016a), freezing was significantly lower in TTX-treated animals (*n* = 9) compared to control conditioned ones (*n* = 12), while it was similar between TTX-treated rats and unpaired (*n* = 10) animals (Figure 1A). As in our previous study (Cambiaghi et al., 2016a), in order to investigate between Te2 and BLA during memory retrieval, we analyzed LFP in Te2 and BLA. An interval of 2 s at the onset of the first CS was analyzed for each animal in conditioned, TTX-injected and unpaired rats (Figure 2B). In fact, our previous study showed that Te2-to-BLA synchronization occurred during the initial 2 s at the onset of the first CS, whilst it was not present the last 2 s of the first CS presentation (Cambiaghi et al., 2016a). By comparing the coherence during the CS onset with respect to the preCS, we found higher levels of coherence in the conditioned group in the low-theta (3.01–7 Hz) range (Kruskal-Wallis test, *P* = 0.011), but not in the other frequency bands namely delta (0.5–3 Hz; Kruskal-Wallis test, *P* = 0.982), high-theta (7.01–12 Hz; Kruskal-Wallis test, *P* = 0.098), beta1 (12.01–20 H; Kruskal-Wallis test, *P* = 0.473) and beta2 (20.01–30 Hz; Kruskal-Wallis test, *P* = 0.653) ranges (Figures 2C–F). In animals retrieving long-term memories, the coherence between Te2 and BLA in the low-theta range was significantly enhanced (median, 0.206) with respect to unpaired (median, −0.024; Mann Whitney test, *P* = 0.006) animals. Conversely, in TTX-conditioned rats, there was no difference in coherence (median, −0.037) from unpaired rats (*P* = 0.388), while coherence was significantly lower in TTX-conditioned rats than the conditioned group (*P* = 0.022; Figures 2C,D). These findings show that Te2 blockade upon memory consolidation processes prevented the theta synchrony occurring between this cortex and the BLA during long-term memory retrieval.

### Te2 Blockade during Memory Consolidation Processes Prevents Learning-Evoked Activity Changes in the Te2 and BLA during Memory Retrieval

**Figure 3:**
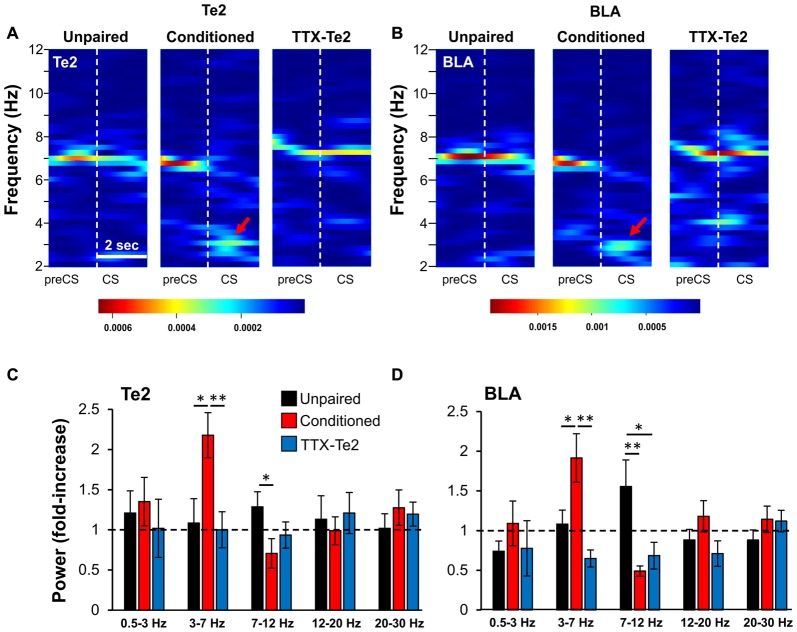
Blocking Te2 during consolidation precludes its activation, as well that of BLA during the early phase of long-term fearful memory recall. Representative spectrograms of Te2 **(A)** and BLA **(B)** activity around the first CS onset (2 s) in an unpaired (*n* = 10), a conditioned (*n* = 12) and a TTX-treated animal (TTX-Te2; *n* = 9). Red arrows indicate the increased activity in the low-theta range. The TTX-treated group showed a low- theta activity similar to the unpaired group in both Te2 **(C)** and BLA **(D)** while conditioned rats displayed an increased low-theta and a decreased high-theta activity in both areas with respect to unpaired and TTX-treated rats (sign-rank, *P* < 0.05). All data are expressed as mean ± SEM. *P < 0.05; **P < 0.01.

We then investigated the effects that blocking Te2 upon memory consolidation could have on the amygdala activity during the retrieval of fearful memories. The neural activity of the BLA was analyzed during retrieval of long-term fear memories in conditioned rats, in those treated with TTX and in unpaired animals. Initially, we analyzed the early phase of memory retrieval (first 2 s), but in order to detect any possible change in activity that could emerge after the cue recall in both Te2 and BLA, we also investigated the entire first CS (8 s). Relative power was analyzed in both Te2 and BLA by dividing the absolute amplitude within the frequency bands delta (0.5–3 Hz), low-theta (3.01–7 Hz), high-theta (7.01–12 Hz), beta1 (12.01–20 Hz) and beta2 (20.01–30 Hz) by the corresponding measures of total amplitude during the pre-CS period (Figures 3A,B). In animals retrieving long-term memories, the early recall phase (2 s) showed significant differences in Te2 (Figure 3C) and BLA (Figure 3D) LFP powers within both the low- and high-theta frequency bands (Kruskal-Wallis, *P* = 0.002 and *P* = 0.015 for BLA, respectively; *P* = 0.009 and *P* = 0.037 for Te2, respectively), but not in the delta, beta1 or beta 2 bands (Kruskal-Wallis, *P* = 0.334, *P* = 0.168 and *P* = 0.482 for BLA, respectively; *P* = 0.384, *P* = 0.735 and *P* = 0.553 for Te2, respectively). However, TTX-injected rats showed no change in the low theta range with respect to unpaired rats in both Te2 (Figure 3C; Mann Whitney test, *P* = 0.706 and *P* = 0.179 respectively) and BLA (Figure 3D; Mann Whitney test, *P* = 0.094 and *P* = 0.053 respectively). The increased activity observed in the 7–12 Hz range in unpaired vs. TTX-Te2 rats might be due to non-associative fear related processes.

**Figure 4:**
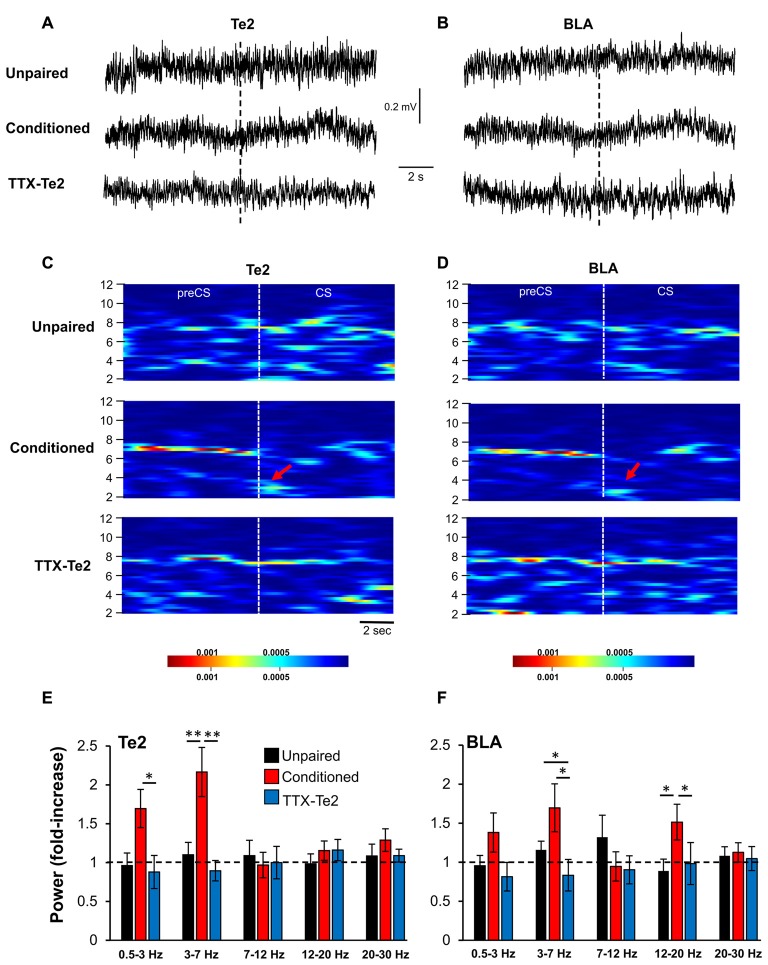
The inactivation of the Te2 cortex abolished LFP activity in BLA during the presentation of the first overall CS. Representative LFP traces recorded in Te2 **(A)** and BLA **(B)** around the first CS (dotted line). Descriptive spectrograms of Te2 **(C)** and BLA **(D)** of the overall first CS (8 s) in unpaired (*n* = 10), a conditioned (*n* = 12) and a TTX-treated rats (TTX-Te2; *n* = 9). Red arrows indicate the increased activity in the low-theta range in the conditioned animal. Low-theta activity in the TTX-treated group did not change with respect to the unpaired group in both Te2 **(E)** and BLA **(F)** (sign-rank, *P* > 0.05), while conditioned rats displayed an increased low-theta activity in both areas with respect to unpaired and TTX-treated rats (sign-rank, *P* < 0.05). All data are expressed as mean ± SEM. *P < 0.05; **P < 0.01.

These data were obtained by analyzing neural activity during the initial 2 s of the first CS and therefore showed that TTX administration into Te2 impaired early memory-related processes during the initial stages of memory retrieval in both Te2 and BLA. However, during the retrieval of the overall learned experience, BLA may display a learning-evoked change due to information encoded in brain sites other than the Te2. To address this issue, we analyzed LFPs of Te2 and BLA during the entire duration (8 s) of the first CS (Figures 4A–D). In both Te2 and BLA, we found a significant difference within the low-theta range among the three groups (Kruskal-Wallis, *P* = 0.001 and *P* = 0.031, respectively), with conditioned rats showing the highest levels of power with respect to both unpaired and TTX-Te2 groups (Mann Whitney test, *P* < 0.05 in both instances; Figures 4E,F). No differences were detected among the three groups in the high-theta band (Kruskal-Wallis, *P* = 0.468 and *P* = 0.914, respectively; Figures 4E,F). In the Te2, a significant difference was observed in the delta range (Kruskal-Wallis, *P* = 0.046), in which conditioned rats had an increased power with respect to both the other groups (Mann Whitney test, *P* < 0.05 in both instances; Figure 4E). In addition, in BLA we found that even in the 12–20 Hz range (Kruskal-Wallis, *P* = 0.034) conditioned rats presented an increased power with respect to unpaired and TTX-Te2 groups (Mann Whitney test, *P* = 0.029 and *P* = 0.027, respectively; Figure 4F).

### Te2 Blockade Hampered the Expression of zif268 Immediate Early Gene in the Individual Lateral, Basal and Central Nuclei during Memory Retrieval

**Figure 5:**
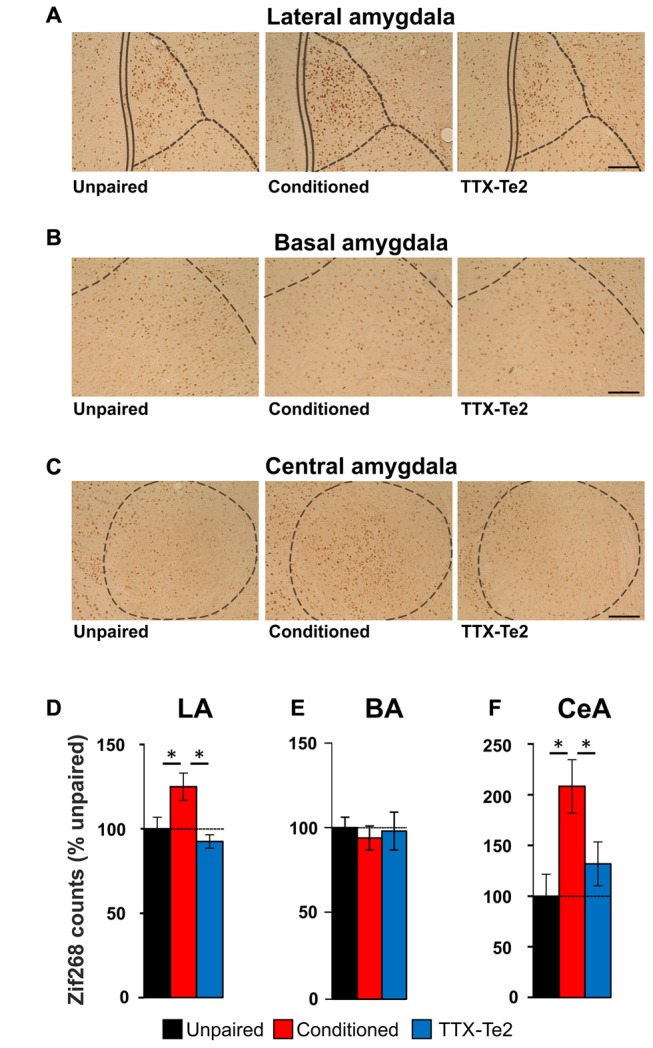
The activity of individual lateral (LA), basal (BA) and central (CeA) amygdala nuclei after fearful memory recall. Photomicrography of zif268 staining in the LA **(A)**, BA **(B)** and CeA **(C)** nuclei of the amygdala in unpaired (*n* = 12), conditioned (*n* = 14) and TTX-injected conditioned (*n* = 12) rats. Scale bars, 150 μm. **(D)** LA activity was enhanced after long-term memory recall mainly in conditioned animals (Newman-Keuls test, *P* < 0.05) but not in those that received TTX after training (Newman-Keuls test, *P* > 0.05). **(E)** BA activity was similar among the three groups (One-way ANOVA). **(F)** zif268 staining in the CeA was enhanced by fearful memory retrieval only in conditioned rats (Newman-Keuls test, *P* < 0.05). All data are mean ± SEM. *P < 0.05.

Our data suggest that blocking memory consolidating processes in the Te2 prevents changes in the BLA activity which may occur during memory retrieval processes. To better address this issue, we employed an alternative method based on the expression of the immediate early gene zif268. Immediate early genes are required for synaptic plasticity and are used as an index of neuronal activation (Frankland and Bontempi, 2005; Sacco and Sacchetti, 2010; Lesburguères et al., 2011; Kwon et al., 2012; Grosso et al., 2017). Among immediate-early genes, zif268 expression has been associated with long-term plasticity that occurs during memory retrieval (Frankland et al., 2004; Kwon et al., 2012; Xie et al., 2014; Grosso et al., 2017). Accordingly, several recent studies have shown changes in zif268 expression in the sensory cortex following emotional memory recall (Hall et al., 2001; Maviel et al., 2004; Sacco and Sacchetti, 2010; Kwon et al., 2012). By employing this technique, previous studies have shown that the activity of LA and central (CeA) amygdala nuclei is enhanced after the retrieval of long-term fearful memories (Sacco and Sacchetti, 2010; Kwon et al., 2012). Therefore, we tracked the expression of zif268 proteins induced in the LA, BA and CeA due to recall of fearful memories in unpaired group (*n* = 12), conditioned animals (*n* = 14) and in animals that received TTX in Te2 after training (*n* = 12; Figures 5A–C). In LA, memory recall produces an increase in zif268 protein levels in conditioned subjects compared to unpaired ones but not in TTX-treated rats (LA, unpaired, 26.82 ± 1.82; conditioned, 33.51 ± 2.21; TTX-Te2, 24.81 ± 1.12; *F*_(2,37)_ = 6.50, *P* = 0.004). Remarkably, the TTX-treated group did not differ from the unpaired one (Newman-Keuls test, *P* > 0.05; Figures 5A,D). These data suggest that LA processes require information processed early on in the cortex. No differences were detected among conditioned, TTX-treated and unpaired groups in zif268 protein expression level in the BA (BA, unpaired, 10.14 ± 0.61; conditioned, 10.00 ± 1.06; TTX-Te2, 9.59 ± 0.70; *F*_(2,37)_ = 0.10; *P* = 0.897; Figures 5B,E). The similarity between naïve and conditioned animals suggests that BA may be not involved in the conditioned freezing response, in line with previous studies showing that blockade of this site did not affect conditioned freezing to auditory CSs (Killcross et al., 1997; Amorapanth et al., 2000; Herry et al., 2008). Conversely, this nucleus may be more prominently required for the active avoidance of threats (Killcross et al., 1997; Amorapanth et al., 2000; Herry et al., 2008).

We finally repeated the analysis of zif268 expression in the CeA of unpaired, conditioned and TTX-injected rats. We found that an increase in zif268 protein levels in conditioned subjects compared to unpaired ones, but not in TTX-treated rats (CeA, unpaired, 0.92 ± 0.19; conditioned, 1.91 ± 0.24; TTX-Te2, 1.21 ± 0.19; *F*_(2,37)_ = 5.68, *P* = 0.005). Again, the TTX-conditioned group did not differ from the unpaired one (Newman-Keuls test, *P* > 0.05; Figures 5C,F).

Taken together, these results show that the reversible blockade of Te2 cortex during memory consolidation processes prevents the enhancement of zif268 expression that normally occurs in most individual amygdala nuclei during memory retrieval.

## Discussion

In the present study we showed that the blockade of Te2 activity during the consolidation processes of auditory fearful memories prevents the increase in both LFP activity and zif268 early gene expression that normally occur in the amygdala during the retrieval of remote memories.

The fact that Te2 was inactivated during the consolidation process in a reversible way and memory retention was tested with functionally intact brain structures allows excluding several alternative interpretations. First, the observed effects cannot be due to the inability of processing the auditory stimuli nor to the transfer of this information to the amygdala or other sites during memory retrieval. For a similar reason, we can also rule out that during the memory retention trial, the Te2 was unable to retrieve memories allocated in the amygdala or elsewhere. Therefore, we proposed that the Te2 is essential for consolidating long-term fearful memories and that in the absence of this process the amygdala is unable to support memory retention and to display memory-related activity, at least the activity that can be detected through LFP and early gene expression analyses. In other words, our data uncovered that when the Te2 cortex is blocked during memory consolidation processes, any other inputs that arrive to the amygdala during memory retrieval cannot provide information sufficient to recruit amygdala nuclei so as to elicit fear-related responses. Finally, we can exclude that TTX administration in Te2 determined a general disturbance of the whole activity of LA. In fact, TTX administration in Te2 1 day after training did not affect the retention of recent memories (Grosso et al., 2015b; Cambiaghi et al., 2016b) whereas an interference with memory consolidation in BLA affected these memories (Sacchetti et al., 1999a; Wilensky et al., 1999).

The amygdala received information also from the auditory thalamic nuclei. The overarching “standard” hypothesis postulates that sensory stimuli can reach the amygdala, and particularly its LA nucleus, through a direct subcortical pathway, which bypasses the sensory cortex and that can support fear reactions *per se* (LeDoux, 2000). Our data do not exclude that during the retrieval of auditory fearful memories, information on the auditory stimuli was able to reach the amygdala through the thalamus and the cortex. However, our data highlighted that, in the absence of auditory cortex during the consolidation phase, auditory stimuli information carried by the auditory thalamus was not sufficient to activate the amygdala and elicit conditioned fear-related reactions. An alternative possibility may be that TTX inactivation of Te2 decreased thalamic activity upon memory consolidation and consequently thalamic projections to the amygdala. Although we cannot exclude this possibility, we should mention that a previous study demonstrated that the blockade of auditory thalamus during memory consolidation did not affect memory retention (Sacchetti et al., 1999a). Indeed, irreversible lesions of the thalamic projections to the amygdala caused severe but incomplete deficits during memory retrieval, while lesions of the thalamo-cortico-amygdala connectivity completely abolished fear memories (Boatman and Kim, 2006). This suggests that the thalamo-cortico-amygdala route is the principal auditory CS pathway when the brain is intact during the retrieval of fear memories.

Our electrophysiological recordings did not enable us to determine whether this pattern activity was restricted to a specific Te2 layer or widespread throughout all six cortical laminae. In fact, previous studies have shown that the retrieval of fearful memories engages all cortical layers (Cho et al., 2016; Grosso et al., 2017). In particular, by measuring zif268 expression after the recall of fearful memories, layers 2 and 3 showed the wider increment across the entire extension of the Te2 (Grosso et al., 2017), in line with the idea that these superficial laminae are recruited by associative processes (Frankland et al., 2004; Maviel et al., 2004; Lesburguères et al., 2011). Furthermore, in Te2 superficial layers, we discovered “associative value-coding” neurons whose activity signals the affective value assigned to auditory stimuli (Grosso et al., 2015b).

Previous studies have shown that during the retrieval of auditory memories, there is an interplay between PF and BLA (Likhtik et al., 2014; Stujenske et al., 2014; Do-Monte et al., 2015; Karalis et al., 2016). It has also been shown that early gene activity was enhanced following remote fear memory retrieval (Do-Monte et al., 2015) and PF inactivation impaired fearful memory retention (Corcoran and Quirk, 2007; Do-Monte et al., 2015), and that the presentation of CSs previously paired to aversive events is associated with an enhanced connectivity between PF and BLA in the theta frequency range (Likhtik et al., 2014; Stujenske et al., 2014; Karalis et al., 2016). It has therefore been hypothesized that fearful memories are stored in this PF-BLA connectivity (Dejean et al., 2015; Do Monte et al., 2016). In this framework, our data suggested that information encoded in PF or in the PF-BLA pathway cannot support the functional absence of memories stored in the auditory cortex. Alternatively, it is possible that PF itself requires information encoded at the level of the auditory cortex and, therefore, in the absence of Te2 memory consolidation processes, the PF cannot recruit amygdala nuclei. Interestingly, a recent study showed that optogenetic induction of low theta (around 4 Hz) activity in the PF synchronized PF-BLA activity and elicited freezing in naïve animals, which had never previously undergone aversive experiences (Karalis et al., 2016). It is tempting to speculate that learned information about the CS-US association is encoded through the connectivity between the auditory cortex and the BLA, whereas the PF-BLA interplay regulates the expression of fear-related behaviors, such as freezing, to learned threat stimuli. In the absence of the Te2-BLA dialog, the PF-BLA pathway should not produce any fear responses. Future studies should investigate these alternative possibilities.

Many studies have shown that the auditory cortex, whether primary or higher order, is essential for associating complex tones to emotional events (LeDoux, 2000; Letzkus et al., 2011; Yang et al., 2016). Conversely, the involvement of these cortices in the association of simple tones with US is more controversial (LeDoux, 2000). More recently, however, it was shown that the higher order auditory cortex plays a crucial role in the long-term storage/retrieval of this association in mice (Cho et al., 2016), rats (Sacco and Sacchetti, 2010; Grosso et al., 2015b; Cambiaghi et al., 2016b) and humans (Apergis-Schoute et al., 2014). The present study fully supports this view, by showing that changes in activity normally seen in individual LA, BA and CeA nuclei during the retrieval of long-term memories are dependent on Te2 participation to memory consolidation processes, and in the absence of this information, any other memory traces allocated elsewhere cannot support amygdala processes and memory retention.

## Author Contributions

MC and BS designed the experiments; MC and AR performed behavioral experiments and analyzed the data. LM performed immunohistochemical experiments and analyzed the data. MC performed electrophysiological experiments and analyzed the data. MC and BS drafted and revised the article.

## Conflict of Interest Statement

The authors declare that the research was conducted in the absence of any commercial or financial relationships that could be construed as a potential conflict of interest.
